# Navigating Perinatal Challenges: A Comprehensive Review of Cholestasis of Pregnancy and Its Impact on Maternal and Fetal Health

**DOI:** 10.7759/cureus.58699

**Published:** 2024-04-21

**Authors:** Mohammed Irfan Abdul Waheed, Arpita Jaiswal, Seema Yelne, Varsha Nandanwar

**Affiliations:** 1 Medicine, Jawaharlal Nehru Medical College, Datta Meghe Institute of Higher Education and Research, Wardha, IND; 2 Obstetrics and Gynaecology, Jawaharlal Nehru Medical College, Datta Meghe Institute of Higher Education and Research, Wardha, IND; 3 Nursing, Shalinitai Meghe College of Nursing, Datta Meghe Institute of Higher Education and Research, Wardha, IND

**Keywords:** perinatal outcomes, obstetric cholestasis, management strategies, maternal-fetal complications, bile acid metabolism, intrahepatic cholestasis of pregnancy (icp)

## Abstract

Cholestasis of pregnancy (CP), or intrahepatic CP (ICP), represents a condition peculiar to pregnancy, marked by impaired bile acid flow and consequent accumulation in the maternal bloodstream. Primarily emerging in the third trimester, CP is linked with considerable risks to both the mother and fetus, including heightened incidences of preterm birth, fetal distress, and stillbirth, alongside maternal complications such as intense pruritus and liver dysfunction. Despite its clinical significance, the etiology of CP, which involves genetic, hormonal, and environmental factors, remains partially understood. This comprehensive review delves into the physiology and pathophysiology of CP, outlines its clinical manifestations and diagnostic criteria, and discusses the associated maternal and fetal complications. Furthermore, it evaluates current management strategies, prognostic implications, and potential long-term effects on maternal and child health. It also explores future research directions, emphasizing the need for advancements in understanding the pathophysiology of CP, developing novel therapeutic interventions, and improving risk stratification models. By offering a thorough overview of CP, this review aims to enhance clinical awareness, guide management practices, and identify areas requiring further investigation, ultimately contributing to better health outcomes for affected women and their babies.

## Introduction and background

Cholestasis of pregnancy (CP), also known as intrahepatic CP (ICP) or obstetric cholestasis, is a liver disorder unique to pregnancy. It is characterized by impaired bile flow, leading to elevated levels of bile acids in the maternal bloodstream. This condition typically manifests during the third trimester and is associated with significant maternal and fetal complications [1]. The recognition of CP dates back several centuries, with early descriptions of its clinical manifestations and associated risks. However, it was only in the latter half of the twentieth century that significant advancements were made in understanding its pathophysiology and management [2].

CP poses serious risks to both maternal and fetal health, including increased rates of preterm birth, fetal distress, and stillbirth [3]. Moreover, maternal complications such as pruritus, sleep disturbances, and liver dysfunction can significantly impact the quality of life during pregnancy and beyond. Given its potential severity, a comprehensive understanding of this condition is essential for effective management and improved outcomes [4]. This review aims to provide a comprehensive overview of CP, focusing on its pathophysiology, clinical manifestations, maternal and fetal complications, management strategies, prognosis, and future research directions. By synthesizing current knowledge and highlighting areas of uncertainty, this review aims to contribute to the understanding and management of CP, ultimately improving outcomes for affected women and their infants.

## Review

Physiology and pathophysiology of CP

Normal Bile Acid Metabolism

In normal bile acid metabolism, the liver plays a pivotal role in synthesizing bile acids from cholesterol. These bile acids are indispensable for facilitating the movement of bile through and out of the liver and are essential for the digestion and absorption of fats and fat-soluble vitamins in the intestines. They act as natural detergents, aiding in the breakdown of fats and vitamins from the diet. Bile acid synthesis involves several sequential steps, each necessitating specific enzymes. Mutations in genes controlling these enzymes can lead to bile acid defects, inherited as autosomal recessive traits, resulting in the incapacity to produce normal bile acids, precipitating liver disease and potentially impacting other organs such as the brain and nervous system [5,6]. On average, the liver synthesizes approximately 200 to 600 mg of bile acids daily, with a comparable amount secreted and excreted daily by an average man. Bile acids are conjugated with amino acids to form sodium salts, enhancing their solubility at physiological pH. Stored in the gallbladder, bile acids are released into the intestinal tract post-meals to facilitate fat absorption. Most bile acids are reabsorbed in the ileum and recycled back to the liver via the enterohepatic circulation, ensuring a constant bile acid pool size. Bile acids that spill into the systemic circulation are eliminated in urine [5,7].

Altered Bile Acid Dynamics in Pregnancy

During pregnancy, alterations in bile acid dynamics, particularly in ICP, result in significant changes in bile acid synthesis, circulation, and their effects on maternal and fetal health. Throughout gestation, there is a notable increase in serum bile acid levels, with some women surpassing standard reference ranges, which can trigger the onset of ICP. Notably, the incidence of ICP is higher in multiple pregnancies compared to singleton pregnancies, and elevated maternal serum bile acid concentrations can lead to the accumulation of bile acids in fetal circulation, thereby increasing the risk of adverse fetal outcomes such as fetal distress, stillbirth, and perinatal mortality [2,8].

The interplay among reproductive hormones, including estradiol, progesterone, and sulfated progesterone metabolites, intricately influences maternal and fetal bile acid homeostasis during pregnancy. Sulfated progesterone metabolites have emerged as crucial regulators of bile acid secretion and homeostasis through the farnesoid X receptor (FXR) signaling pathway. Notably, allopregnanolone sulfate and epiallopregnanolone sulfate have been implicated in maternal and fetal bile acid homeostasis, demonstrating correlations with bile acid levels in maternal and fetal serum. Moreover, these metabolites exhibit antagonist activity on FXR-mediated bile acid homeostasis, suggesting their potential to mitigate the adverse effects of elevated bile acids during pregnancy [8]. Understanding the altered dynamics of bile acids in pregnancy, particularly in conditions such as ICP, is imperative for identifying potential therapeutic targets and interventions to manage the risks associated with disrupted bile acid metabolism. Further investigation into the mechanisms underlying these alterations holds promise in elucidating the pathogenesis of cholestasis during pregnancy and facilitating the development of targeted strategies to safeguard maternal and fetal well-being.

Genetic and Environmental Factors

ICP is widely recognized as a genetic disorder, with individuals harboring a genetic predisposition to its development [9,10]. Even without a familial history of ICP, inheriting a single copy of a gene associated with the condition can elevate one's susceptibility to its onset. This genetic diversity contributes to the varied clinical presentations observed among affected individuals. Extensive research has pinpointed numerous gene mutations implicated in ICP, and its familial clustering strongly indicates a genetic predisposition [9]. Notably, mothers and sisters of individuals with ICP face an elevated risk of experiencing the disorder, underscoring its hereditary nature. While genetic factors play a significant role, environmental influences also contribute to the etiology of ICP. Despite a limited understanding, various environmental factors, including dietary patterns and seasonal variations, have been implicated in its causation [11]. However, the precise mechanisms underlying these environmental influences remain elusive, posing challenges in predicting the likelihood of developing ICP in any given pregnancy. Although isolated studies have tentatively associated ICP with pregnancies occurring during winter months and selenium deficiency, conclusive evidence regarding the impact of diet on ICP risk is lacking [11]. Nevertheless, anecdotal reports suggest that dietary modifications may ameliorate ICP symptoms in some individuals, indicating a potential interplay between environmental factors and the condition.

Hormonal Influences

Hormonal influences are pivotal in the pathogenesis of ICP. Throughout pregnancy, hormonal fluctuations, particularly heightened levels of estrogen and progesterone, can impact bile flow within the liver. These hormonal changes may precipitate a reduction in bile flow, thereby contributing to the development of cholestasis. Additionally, the hormone human chorionic gonadotropin (hCG) has been implicated in the pathophysiology of ICP, with research indicating a potential correlation between elevated hCG levels and the onset of this condition [1]. The intricate hormonal milieu characteristic of pregnancy can disrupt the normal processes of bile formation and secretion, culminating in cholestasis. Understanding the intricate interplay between hormones and liver function, specifically their effects on bile flow regulation, is paramount in elucidating the pathophysiology of ICP and its ramifications for maternal and fetal health. By comprehending the hormonal influences on hepatic physiology, healthcare professionals can better identify, manage, and mitigate the risks associated with ICP.

Immune System Involvement

The immune system plays a significant role in the pathogenesis and progression of ICP, contributing to its complex disease process. Emerging research highlights the involvement of inflammatory cytokines, such as interleukin-6 (IL-6), in ICP, underscoring their pivotal role in the observed inflammatory response [12,13]. IL-6, in particular, is a sensitive marker of inflammation in ICP, further emphasizing the immune system's integral contribution to the disease mechanism. Additionally, investigations have demonstrated that maternal cholestasis can influence the immune function of offspring, potentially leading to metabolic and immune system alterations in neonates born to mothers with ICP [13]. Fetal exposure to elevated bile acid levels in utero has been associated with an increased susceptibility to inflammatory diseases in offspring, highlighting the profound impact of the maternal immune system on the developing fetus's health. Overall, the immune system's involvement in ICP encompasses a multifaceted interplay of inflammatory cytokines, oxidative stress, and immune system adaptations. These factors collectively contribute to the pathophysiology of ICP and underscore its profound implications for maternal and fetal health. Understanding the intricate relationship between the immune system and ICP holds promise for advancing therapeutic strategies and improving outcomes for affected individuals.

Clinical manifestations and diagnosis

Symptoms and Signs

**Figure 1:**
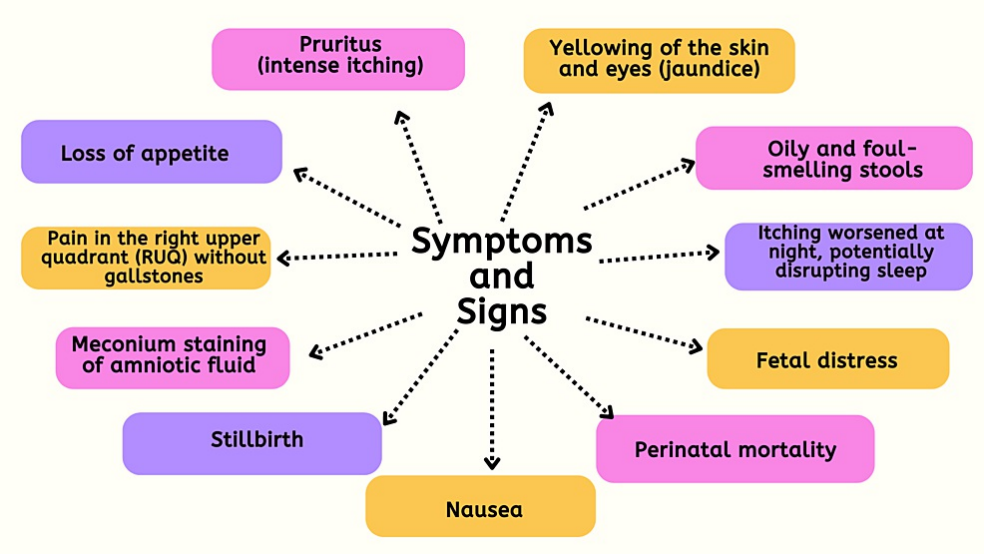
Symptoms and signs of cholestasis of pregnancy The image is created by the corresponding author.

The manifestations of ICP encompass intense itching (pruritus) devoid of a rash, typically localized on the palms of the hands and soles of the feet, but potentially extending to other areas of the body. This itching tends to worsen during the nighttime and may disrupt sleep patterns. Additional symptoms may include yellowing of the skin and eyes (jaundice), feelings of nausea, loss of appetite, the presence of oily and foul-smelling stools, and discomfort in the right upper quadrant (RUQ) of the abdomen in the absence of gallstones. In severe instances, ICP can precipitate complications such as meconium staining of amniotic fluid, fetal bradycardia, and even fetal loss. It is imperative for expectant individuals experiencing these symptoms to promptly seek medical attention for accurate diagnosis and appropriate management [14,15]. Figure 1 illustrates the symptoms and signs.

Laboratory Investigations

**Figure 2:**
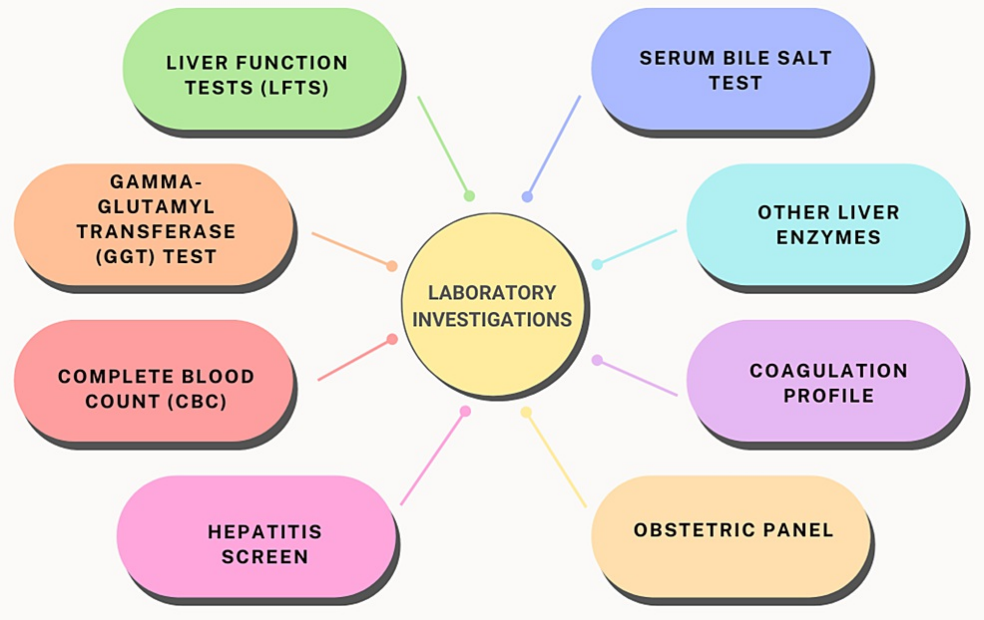
Laboratory investigations for cholestasis of pregnancy The image is created by the corresponding author.

Laboratory investigations for ICP entail blood tests aimed at aiding diagnosis. These tests encompass liver blood tests and a serum bile salt test. Liver blood tests evaluate liver function by assessing various blood properties, particularly liver enzymes such as alanine aminotransferase (ALT) and aspartate aminotransferase (AST). Elevated levels of these enzymes and heightened serum bile salt levels serve as indicators of ICP. Also, gamma-glutamyl transferase (GGT) levels may sometimes be elevated. The most specific diagnostic test for ICP involves the measurement of serum bile salts, which are typically elevated in affected women. Notably, the availability of the serum bile salt test may vary across hospitals, necessitating the sending of samples to specialized facilities for diagnosis. Repeat testing may be warranted if initial results prove inconclusive. Furthermore, it is essential to recognize that women with hepatitis C are at an increased risk of developing ICP during pregnancy, underscoring the significance of comprehensive laboratory investigations in distinguishing between these conditions [16,17]. Figure 2 illustrates laboratory investigations.

Differential Diagnosis

**Figure 3:**
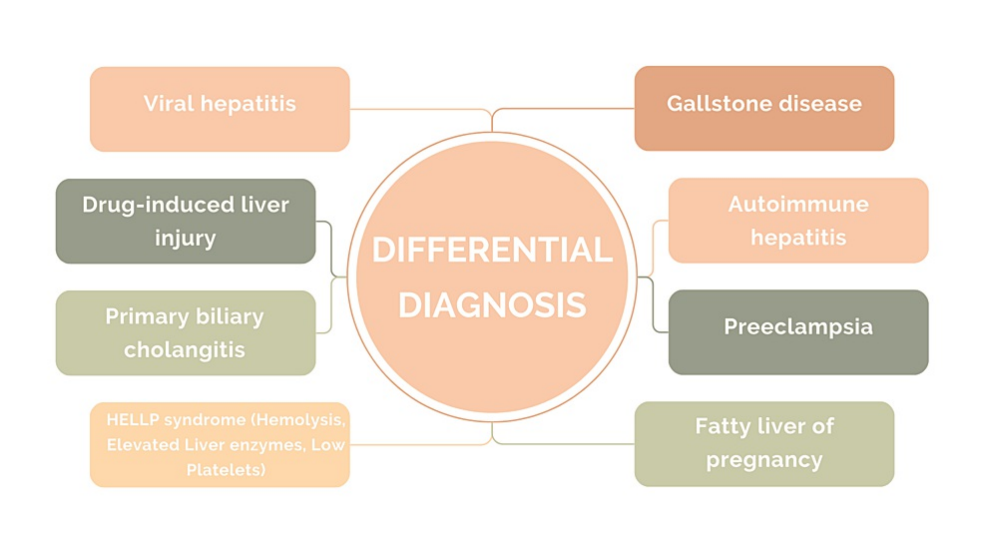
Differential diagnosis of cholestasis of pregnancy The image is created by the corresponding author.

The process of differential diagnosis for ICP necessitates the exclusion of other conditions that might manifest with similar symptoms. Several potential differential diagnoses merit consideration, including viral hepatitis, gallstone disease, drug-induced liver injury, and various liver disorders such as autoimmune hepatitis and primary biliary cholangitis. Additionally, conditions such as preeclampsia, HELLP syndrome (hemolysis, elevated liver enzymes, low platelet count), and fatty liver during pregnancy can also exhibit overlapping symptoms, underscoring the importance of a comprehensive assessment to distinguish ICP from these entities [18,19]. Figure 3 illustrates differential diagnosis.

Diagnostic Challenges

**Figure 4:**
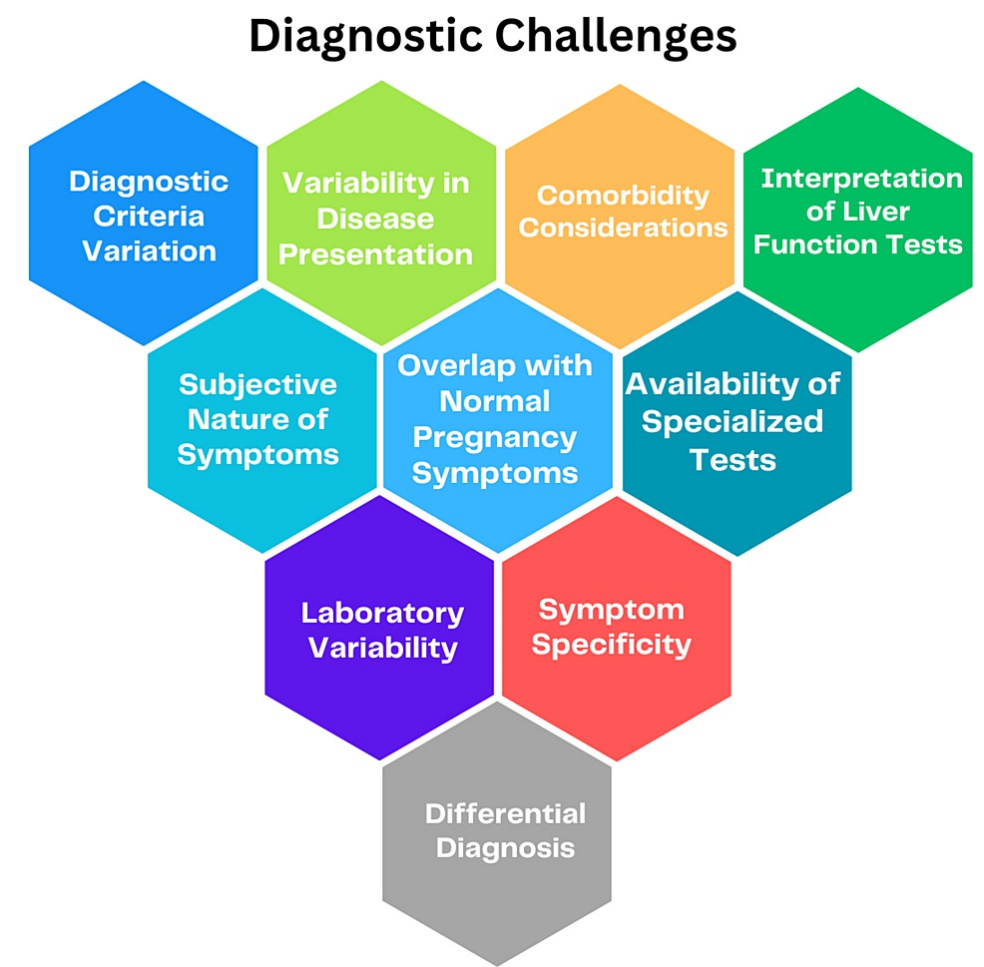
Diagnostic challenges of cholestasis of pregnancy The image is created by the corresponding author.

Diagnostic challenges in ICP may arise due to the complexity of symptoms and the necessity to distinguish ICP from other liver disorders. The diagnosis of ICP primarily relies on symptoms, with a focus on pruritus typically affecting the palms and soles, coupled with elevated bile acid levels. However, pruritus alone lacks specificity for ICP and can manifest in other conditions [20]. Consequently, additional liver function tests, such as ALT and AST, are commonly elevated in ICP but can also be influenced by other liver dysfunctions. Thus, it becomes imperative to rule out alternative causes of liver abnormalities. This underscores the significance of a comprehensive diagnostic approach integrating clinical symptoms and laboratory findings to accurately identify and differentiate ICP from other liver disorders [20]. Figure 4 illustrates diagnostic challenges.

Maternal complications associated with CP

Liver Dysfunction

Liver dysfunction during pregnancy can manifest in various forms, including conditions such as ICP, acute fatty liver of pregnancy (AFLP), and HELLP syndrome (hemolysis, elevated liver enzymes, low platelets). These liver disorders pose significant risks of adverse maternal and fetal outcomes, with AFLP being particularly underdiagnosed and carrying a risk of maternal death if not promptly recognized and managed [21]. Given the potential severity of these conditions, liver dysfunction during pregnancy often necessitates specialized intervention and close monitoring to ensure early diagnosis and appropriate management, thereby safeguarding both maternal and fetal health [22]. When making therapeutic decisions for liver disorders during pregnancy, it is crucial to consider the implications for both the mother and the child. Factors such as the type of liver disease, the degree of impaired liver function, and the timing of delivery play pivotal roles in determining maternal prognosis [22].

Increased Risk of Gallstones

A variety of factors influence the risk of developing gallstones. Key risk factors include obesity, elevated estrogen levels due to pregnancy or hormone therapy, specific ethnic backgrounds such as Native Americans, female gender, advancing age (especially over 60), certain medications like cholesterol-lowering drugs, diabetes, rapid weight loss, and prolonged fasting [23,24]. These factors can contribute to the formation of gallstones by impacting cholesterol levels in bile, gallbladder motility, and bile composition. Women, in particular, face a heightened risk of gallstone development compared to men, with obesity and elevated estrogen levels playing significant roles in gallstone formation [24].

Pruritus and Sleep Disturbances

Pruritus, commonly referred to as itching, and sleep disturbances are significant challenges associated with pregnancy, particularly in conditions such as ICP. Pruritus is a prevalent symptom of ICP, affecting pregnant women with this condition due to elevated bile acid levels [3]. Sleep disruptions during pregnancy are widespread and influenced by various factors, including physical symptoms such as frequent urination, discomfort, and pain, as well as psychologically based symptoms like concerns about the baby, pregnancy, and labor/delivery [25]. These disturbances can have a profound impact on the quality of sleep and overall well-being of pregnant women, underscoring the importance of managing symptoms such as pruritus and addressing sleep disruptions during pregnancy.

Impact on Quality of Life

ICP can profoundly impact the quality of life of pregnant individuals. This condition, characterized by intense itching without a rash, can be distressing and disrupt daily activities due to the discomfort and sleep disturbances caused by the itching. The uncertainty and anxiety surrounding the potential risks to the fetus, such as premature birth and stillbirth, can also lead to emotional distress and affect the overall well-being of pregnant individuals [17]. Moreover, the necessity for regular monitoring through blood tests and potential interventions such as treatment with ursodeoxycholic acid or even premature delivery in severe cases can further add to the physical and emotional burden experienced by pregnant individuals with ICP. The association of the condition with adverse pregnancy outcomes, an increased likelihood of cesarean section, and the need for specialized care from healthcare providers can also contribute to the overall impact on quality of life during pregnancy [2,26].

Psychological Effects

The psychological effects of ICP can be profound, stemming from various sources of stress. These may include the intense pruritus (itching) experienced by affected individuals, the anxiety associated with awaiting a diagnosis, and the emotional impact of being diagnosed with a rare condition late in pregnancy. The persistent itch, coupled with the consequent sleep deprivation, can significantly contribute to psychological distress during pregnancy. Moreover, the shock of receiving a diagnosis of a condition like ICP, which carries risks to both maternal and fetal health, can further exacerbate emotional stress and prompt concerns about the well-being of the baby. Raising awareness about ICP among friends, family, and healthcare providers is paramount to ensure timely diagnosis, appropriate treatment, and support for women experiencing this condition [27]. By fostering understanding and support networks, individuals affected by ICP can receive the necessary care and assistance to navigate the challenges associated with this condition, thereby promoting their overall well-being during pregnancy.

Fetal complications associated with CP

Intrauterine Growth Restriction

Intrauterine growth restriction (IUGR) refers to the inadequate growth of a baby during pregnancy while in the mother's womb. It is characterized by fetal weight estimated to be below the 10th percentile for its gestational age, indicating that the fetus is smaller than expected relative to its age [28,29]. Multiple factors can contribute to the development of IUGR, including placental issues, infections, maternal health conditions such as high blood pressure or diabetes, and lifestyle factors such as smoking or inadequate nutrition [28]. IUGR carries an increased risk of perinatal mortality and morbidity, emphasizing the importance of prompt diagnosis and management to mitigate adverse outcomes [30]. Monitoring fetal growth through techniques such as ultrasounds, blood flow assessments, and non-stress testing plays a critical role in managing IUGR, with the possibility of early delivery depending on the severity of the condition [28]. Overall, IUGR presents risks to both the mother and the fetus, underscoring the importance of vigilant monitoring and appropriate interventions to optimize outcomes for both parties.

Preterm Birth

Preterm birth represents a significant concern associated with ICP. Research findings indicate that ICP poses a substantial risk to the unborn child, elevating the likelihood of preterm delivery [31-33]. Studies suggest that elective early delivery, typically around 35 to 36 weeks of gestation for women with total bile acid values of 100 micromol/L or higher, may be advised to diminish the risk of unexpected fetal demise in cases of ICP [31]. Furthermore, treatment with ursodeoxycholic acid (UDCA) has been shown to reduce the risk of premature birth in mothers with ICP by over 40%, particularly benefiting those with more severe ICP who are at a heightened risk of spontaneous preterm birth [32]. These findings underscore the critical importance of monitoring and managing ICP to mitigate the risk of preterm birth and its associated complications for both the mother and the fetus.

Stillbirth

Stillbirth is a devastating event characterized by the loss of a baby in the womb after 20 weeks of pregnancy. Common causes of stillbirth include maternal or fetal infections, complications related to the placenta or umbilical cord, pregnancy-related complications such as preeclampsia, and genetic abnormalities in the baby [34-37]. Various risk factors contribute to the likelihood of stillbirth, including medical conditions such as obesity and diabetes, advanced maternal age, carrying multiple babies, being of African American descent, a history of previous stillbirth, high blood pressure, diabetes, and other underlying medical conditions [36,38]. Healthcare providers play a crucial role in conducting tests to determine the cause of stillbirth, which not only aids in preventing future occurrences but also offers closure for the parents affected [36]. Coping with the profound grief of stillbirth is a challenging journey, and seeking support, expressing emotions, educating oneself about the grieving process, and allowing sufficient time for healing are vital components of navigating this difficult experience [36].

Respiratory Distress Syndrome

Respiratory distress syndrome (RDS) is a respiratory condition primarily affecting premature infants due to underdeveloped lungs that lack surfactant, a substance crucial for keeping the lungs open for breathing. Unlike bronchopulmonary dysplasia (BPD), another condition affecting premature babies, RDS typically manifests within the first 24 hours after birth. Risk factors for RDS include prematurity, maternal diabetes, cesarean delivery, and instances of asphyxia during birth. In the United States, RDS impacts an estimated 20,000 to 30,000 newborns annually, with a higher incidence observed in infants born at 26 to 28 weeks gestation than those born at 30 to 31 weeks. Complications of RDS range from acute issues like alveolar rupture and infection to chronic complications such as BPD and retinopathy of prematurity (ROP). Treatment for RDS typically involves surfactant replacement therapy and respiratory support. Advances such as antenatal steroids and gentle ventilation techniques have improved outcomes for affected infants [39-41].

Meconium Passage

Meconium passage refers to a newborn passing meconium, the early stool produced before the baby begins feeding on milk or formula. Typically, healthy newborns pass meconium within the first 24 to 48 hours after birth. However, in some instances, meconium can be expelled while the baby is still in the uterus, leading to potential complications such as meconium aspiration syndrome (MAS) if the baby inhales the meconium-stained amniotic fluid. MAS can result in respiratory difficulties in newborns and is linked to various risk factors, including fetal distress, reduced oxygen supply, challenging delivery, maternal hypertension, and placental infection [42,43]. Meconium passage in utero can be influenced by factors like gestational age, fetomaternal stress, infection, and gastrointestinal maturation, with meconium aspiration contributing to MAS, a significant contributor to perinatal mortality [43]. Understanding the mechanisms, consequences, and management of meconium passage is critical in obstetric and neonatal care to prevent and address potential complications associated with meconium aspiration.

Management strategies

General Principles

Managing ICP involves several key strategies to ensure the well-being of both the mother and the fetus. Firstly, regular monitoring and testing are crucial aspects of ICP management. Pregnant women suspected of having ICP should undergo regular monitoring, which includes measuring serum bile acid and liver transaminase levels. Additionally, antenatal fetal surveillance should commence at an appropriate gestational age. This surveillance is guided by the potential need for delivery based on abnormal fetal testing results or the timing of diagnosis if made later in gestation [44]. Secondly, treatment with ursodeoxycholic acid (UDCA) is recommended as the first-line treatment for managing maternal symptoms of ICP. It is advisable to initiate UDCA at the onset of ICP symptoms, even before receiving laboratory results. UDCA has been shown to alleviate pruritus and improve maternal well-being [45].

The decision regarding the delivery timing is a critical aspect of ICP management. Patients with total bile acid levels of 100 μmol/L or higher should be offered delivery at 36 0/7 weeks of gestation due to the increased risk of stillbirth around this time. For patients with lower bile acid levels, delivery between 36 0/7 and 39 0/7 weeks of gestation is recommended [45]. Additionally, administering antenatal corticosteroids for fetal lung maturity is advised for patients delivering before 37 0/7 weeks of gestation if not previously administered. This intervention helps reduce the risk of respiratory complications in preterm infants [45]. Lastly, preterm delivery before 37 weeks of gestation should be avoided in patients with a clinical diagnosis of ICP without laboratory confirmation of elevated bile acid levels. This highlights the importance of accurate diagnosis and appropriate management decisions [45]. These general principles encompass key aspects of managing ICP, focusing on monitoring, treatment, delivery timing, and interventions to optimize outcomes for both the mother and the fetus.

Pharmacological Interventions

Several pharmacological interventions have been explored for the treatment of ICP. Ursodeoxycholic acid (UDCA) stands out as a commonly used treatment option. Studies have evaluated the efficacy of UDCA in reducing maternal symptoms such as pruritus and improving biochemical alterations associated with ICP. Randomized controlled trials have demonstrated the potential benefits of UDCA in managing ICP [46,47]. Another pharmacological intervention under investigation is S-adenosylmethionine (SAMe). However, the evidence regarding its effectiveness in managing ICP is limited, necessitating further research to establish its role in treating the condition [48].

Activated charcoal, guar gum, and cholestyramine bind bile acids in the intestine, forming part of the pharmacological interventions explored for ICP treatment. Clinical trials have investigated their efficacy in managing ICP symptoms and improving maternal and fetal outcomes [48]. Chinese herbal medicines, including yinchenghao decoction (YCHD) and salvia, have also been studied for their potential benefits in treating ICP. These herbal remedies constitute a diverse array of pharmacological interventions explored to address the symptoms and complications of ICP [48]. Rifampicin has been considered for use in severe cases of early-onset ICP. The TURRIFIC study, a multicenter international randomized controlled trial, aims to evaluate the efficacy of rifampicin compared to UDCA in severe early-onset ICP. This study seeks to assess the impact of rifampicin on pruritus and other clinical outcomes in women with severe ICP [48].

Non-pharmacological Interventions

Dietary modifications play a significant role in alleviating symptoms associated with ICP. Recommendations often include adopting a diet low in saturated fats and rich in fiber, while also avoiding spicy foods and alcohol. Consuming small, frequent meals can aid in managing symptoms like nausea and indigestion [2]. Monitoring bile acid levels and liver function tests is essential for managing ICP effectively. Lifestyle adjustments are also crucial, such as refraining from hot baths, using mild soaps, and opting for loose, breathable clothing to reduce itching. Maintaining a cool environment and applying moisturizers can provide additional relief from pruritus [49]. Emotional support and counseling play a vital role in managing ICP, as the condition can be stressful and impact mental well-being. Support groups or counseling sessions can offer valuable assistance to women coping with the challenges of ICP during pregnancy [49]. Monitoring fetal health is paramount in managing ICP. Regular fetal monitoring through ultrasounds and non-stress tests helps assess the well-being of the fetus. Close monitoring enables early detection of any signs of distress, guiding decisions regarding the timing of delivery to prevent adverse outcomes [50]. Identifying and avoiding potential triggers that exacerbate symptoms, such as hot weather, tight clothing, or certain skincare products, can aid in managing pruritus and discomfort associated with ICP [2].

Timing and Mode of Delivery

In considering the timing of delivery for patients with ICP, several factors come into play. A retrospective cohort study revealed that preterm delivery in ICP patients correlated with various factors, including higher bile acid levels, presence of hepatitis C, earlier diagnosis, lower Apgar scores, increased rates of hyperbilirubinemia, NICU admission, and longer NICU stays. Notably, induction before 37 weeks gestation was associated with higher rates of cesarean section, whereas vaginal delivery was more prevalent for inductions at or after 37 weeks [51]. Recent guidance from the Society for Maternal-Fetal Medicine suggests delivery at 36 weeks gestation for patients with serum bile acid levels exceeding 100 μmol/L. For patients with total bile acid levels below 100 μmol/L, delivery between 36 0/7 and 39 0/7 weeks of gestation is recommended [52]. Additionally, a study found that induction of labor at either 38 or 40 weeks of gestation for women with mild ICP yielded comparable obstetrical outcomes [53]. Regarding the mode of delivery, the Society for Maternal-Fetal Medicine recommends administering antenatal corticosteroids for fetal lung maturity to patients delivering before 37 weeks of gestation if not previously administered. However, preterm delivery before 37 weeks is not recommended for patients with a clinical diagnosis of ICP without laboratory confirmation of elevated bile acid levels [44]. The study underscores that advanced ICP disease often leads to spontaneous preterm labor before a scheduled induction, underscoring the complexity of managing the timing and mode of delivery in ICP cases [51].

Management of Pruritus and Itch

Managing pruritus effectively involves a systematic approach addressing various aspects of the condition. The initial step is identifying the underlying cause, which could stem from a wide range of factors such as dry skin, dermatological conditions like eczema, systemic diseases including renal or hepatic issues, infections, drug reactions, or malignancies [54]. Once the cause is determined, nonpharmacologic measures are pivotal in managing pruritus. These measures encompass frequent moisturization, avoiding rough clothing or overheating, maintaining short and clean fingernails, and cautiously using vasodilators if they trigger itching [55]. Topical treatments serve as an essential component in providing symptomatic relief. These may include wet dressings, calamine lotion, menthol/camphor lotion, local anesthetics, emollients, mild topical corticosteroids, and calcineurin inhibitors, particularly for inflammatory skin conditions [56]. In cases of severe pruritus disrupting sleep, systemic therapy may be warranted. Antihistamines can alleviate itch, while other medications with sedative effects might be prescribed. Treatment approaches may vary depending on the underlying cause of pruritus [57]. Further investigations may be necessary for patients with chronic pruritus to tailor treatment to the specific cause. When the cause remains elusive or refractory, alternative options such as capsaicin, calcineurin inhibitors, naltrexone, pregabalin, ultraviolet therapy, and cyclosporine might be considered [58]. Given the multifaceted nature of pruritus, an interdisciplinary approach involving collaboration among healthcare professionals is often essential. Dermatologists, internists, and specialists in liver or kidney diseases may need to work together to address the root cause and provide appropriate treatment [59].

Prognosis and long-term implications

Short-Term Outcomes

Studying the impact of increased intracranial pressure (ICP) on short-term outcomes among traumatic brain injury (TBI) patients is a critical research area. Evidence indicates that heightened levels of ICP correlate with poorer outcomes, including respiratory complications, infections, prolonged stays in the intensive care unit, and extended periods of mechanical ventilation [60,61]. Given these associations, closely monitoring ICP becomes crucial for early identification and management of complications, potentially improving patient outcomes. Researchers underscore the significance of developing noninvasive methods for ICP monitoring, reassessing treatment triggers, and advancing targeted therapies to address the challenges posed by elevated ICP in TBI patients [60]. Moreover, questions have been raised regarding the effectiveness and safety of ICP monitoring in guiding therapies for TBI patients, highlighting the necessity for further investigation to optimize the utility of ICP monitoring and enhance short-term outcomes in this population [61]. In summary, effectively managing ICP levels and employing appropriate monitoring strategies play pivotal roles in bolstering short-term outcomes for individuals grappling with traumatic brain injuries.

Long-Term Health Implications for Mother and Child

The provided sources indicate that the long-term health implications for mothers and children are extensive and interconnected. Maternal health throughout pregnancy and postpartum plays a pivotal role in shaping the health outcomes of both the mother and the child over the long term. Studies have shown that poor maternal physical and mental health during pregnancy or up to 15 months post-childbirth can lead to adverse effects on the overall health, prevalence of chronic conditions, and physical health outcomes of children, underscoring the intricate relationship between maternal and child health [62]. Furthermore, the timing of childbearing and parity have been associated with various health outcomes for mothers later in life. Early childbearing has been linked to heightened risks of limitations in daily activities and increased levels of depressive symptoms. In contrast, late childbearing is associated with a greater incidence of depressive symptoms [63]. Additionally, pregnancy and childbirth can contribute to or exacerbate conditions such as depression, urinary and anal incontinence, and sexual dysfunction, leading to medium- and long-term complications that are often overlooked in maternal healthcare. This underscores the necessity for a more comprehensive approach to postpartum care, extending beyond the traditional six-week period [64]. In essence, the long-term health implications for mothers and children are influenced by many factors, including maternal physical and mental well-being during and after pregnancy, the timing of childbearing, parity, and chronic conditions. Recognizing and addressing these factors are vital in promoting mothers' and children's long-term health and well-being.

Recurrence Risk in Subsequent Pregnancies

The recurrence risk in subsequent pregnancies varies depending on the specific pregnancy-related condition. For instance, in the case of pre-eclampsia, research suggests that the risk of recurrence is approximately 15% for women who experienced pre-eclampsia in one previous pregnancy, and it rises to around 30% when two pregnancies are affected [65]. Similarly, for stillbirth, women who experienced a stillbirth in their first pregnancy have a heightened risk of subsequent stillbirth, with an adjusted hazard ratio ranging from 1.86 to 2.72 compared to women who had a live birth in their first pregnancy [66]. Concerning fetal growth restriction (FGR), women with a previous pregnancy affected by FGR face a recurrence risk ranging from 20% to 30% in subsequent pregnancies. Currently, limited effective preventive strategies are available to mitigate this risk [66].

Additionally, for gestational diabetes mellitus (GDM), the risk of recurrence in subsequent pregnancies varies, with studies indicating a 52% increased risk and a recurrence rate of GDM as high as 35% in subsequent pregnancies [67]. These findings underscore the significance of monitoring and managing these conditions in subsequent pregnancies to mitigate the recurrence risks. By closely monitoring at-risk individuals and implementing appropriate management strategies, healthcare providers can help optimize outcomes and reduce the likelihood of recurrence in subsequent pregnancies.

Future directions and research opportunities

Advancements in Understanding Pathophysiology

Recent advancements in understanding the pathophysiology of ICP, also known as obstetric cholestasis, have significantly contributed to the field. Studies have elucidated that ICP is characterized by elevated levels of circulating bile acids and liver dysfunction, which are implicated in adverse fetal outcomes like preterm labor and stillbirth [68]. Key to this understanding is the recognition of inflammatory mechanisms triggered by heightened bile acid levels, which underlie the pruritic symptoms commonly experienced during pregnancy [69]. This newfound comprehension of the disease's underlying mechanisms has provided crucial insights into potential targets for treatment and strategies to enhance the care of pregnant women affected by ICP [68]. Moreover, recent research has identified genetic predispositions and mutations in hepatobiliary transport proteins, including multidrug resistance protein 3 (MDR3) and bile salt export pump (BSEP), as significant factors in ICP development, underscoring the genetic component in the disorder's pathophysiology [2]. Regular monitoring of serum total bile acid (TBA) levels throughout pregnancy is deemed essential, with concentrations exceeding 40 μmol/L indicating severe ICP and a heightened risk of fetal complications [2]. Therapeutic interventions aim to alleviate clinical symptoms, normalize maternal biochemistry, and prevent fetal complications, with ursodeoxycholic acid administration often forming a cornerstone of pharmacological treatment [2]. These advances in understanding the pathophysiology of ICP have deepened our comprehension of the condition and paved the way for more targeted and effective management strategies. The significance of ongoing research in unraveling the complexities of ICP cannot be overstated, as it can further enhance outcomes for mothers and their babies affected by this condition.

Novel Therapeutic Targets

Recent research has unveiled promising avenues for advancing the therapeutic landscape of ICP, shedding light on novel targets and diagnostic approaches. One such avenue involves targeting gut microbiota-associated bile salt hydrolases, which have emerged as potential therapeutic targets for ICP due to their involvement in bile acid metabolism [68]. By modulating these enzymes, it may be possible to intervene in the dysregulated bile acid metabolism characteristic of ICP, offering new avenues for therapeutic intervention. Metabolomics and proteomics have also emerged as promising fields for identifying biomarkers associated with ICP. Biomolecules such as peroxisomal acyl-CoA oxidase 1 (ACOX1), L-palmitoylcarnitine, and glycocholic acid, identified in placental tissue through metabolomic and proteomic analyses, hold promise as diagnostic and predictive biomarkers for ICP [70]. Harnessing these biomarkers could enhance ICP diagnosis accuracy and facilitate disease progression prediction, enabling timely and targeted therapeutic interventions.

Furthermore, genetic mutations in ABC transporter genes have been identified as potential targets for therapeutic interventions in ICP. These mutations, which influence bile salt transport and play a significant role in the pathogenesis of ICP, represent an intriguing avenue for precision medicine approaches in managing this condition [71]. Targeting specific genetic aberrations may be possible to modulate bile salt transport and mitigate the pathological processes underlying ICP, offering personalized therapeutic strategies tailored to individual patients.

Predictive Models for Risk Stratification

In advancing risk assessment methodologies, a shift toward continuous risk predictions has garnered attention for its potential to enhance decision-making processes at an individual level. Unlike traditional risk group classifications, continuous risk estimates provide a more nuanced understanding of risk, avoiding the oversimplification associated with threshold-based categorizations. By offering granular insights into risk profiles, continuous risk predictions enable clinicians to tailor interventions and management strategies more precisely, optimizing patient care and outcomes [72]. Developing predictive risk stratification tools represents a significant stride toward proactive healthcare management. These tools leverage multidimensional risk factors and predictive models to identify populations at heightened risk for specific health outcomes, such as new-onset stroke. By stratifying individuals into distinct risk groups based on comprehensive risk profiles, these tools empower healthcare providers to allocate resources efficiently and implement preventive measures tailored to each patient's unique risk profile [73]. Risk stratification tools play a multifaceted role in healthcare delivery, offering valuable support across various clinical contexts. Beyond informing clinical care decisions, these tools assist in patient selection for targeted interventions or clinical trials, facilitating the delivery of personalized treatment approaches. Moreover, risk stratification enables healthcare providers to anticipate hospitalization duration and predict discharge disposition, contributing to resource optimization and streamlining healthcare delivery processes in diverse healthcare settings [74].

## Conclusions

In conclusion, CP represents a significant concern due to its potential to cause severe maternal discomfort and substantial risks to the fetus, including preterm delivery and stillbirth. This review has provided a thorough overview of the condition, from its pathophysiological underpinnings and clinical presentation to the management strategies to mitigate its impacts. Recognizing the symptoms and implementing appropriate treatment protocols, such as administering ursodeoxycholic acid, cannot be overstated. The condition underscores the necessity of a well-coordinated, multidisciplinary approach encompassing obstetricians, hepatologists, and neonatologists to optimize maternal and fetal outcomes. Looking forward, the future of managing CP lies in advancing our understanding through research, improving diagnostic methodologies, and developing more effective treatments. Challenges remain in predicting which pregnancies will be affected by cholestasis and in fully understanding the long-term implications for both mothers and their children. Nevertheless, ongoing research and a commitment to a collaborative care model hold promise for better management of this complex condition, ultimately leading to safer pregnancies and healthier outcomes for mothers and babies alike.
